# Unraveling the Puzzle: The Secret Hideout of Methicillin-Resistant Staphylococcus aureus

**DOI:** 10.7759/cureus.66889

**Published:** 2024-08-14

**Authors:** Fawwad A Ansari, Sheri P Walls, Dylan Hewlett, Priscilla E Biney

**Affiliations:** 1 Internal Medicine, Piedmont Athens Regional Medical Center, Athens, USA

**Keywords:** bacteremia, methicillin resistant staphylococcus aureus (mrsa), sepsis, meningitis, epidural abscess

## Abstract

Methicillin-resistant *Staphylococcus aureus* (MRSA) is a prevalent nosocomial pathogen known for causing severe disseminated infections. Recently, there has been an increase in community-acquired MRSA infections. We present a case of MRSA bacteremia complicated by a cervical epidural abscess. A 63-year-old female with no significant past medical history presented with altered mental status lasting two days. She had recently experienced neck stiffness after lifting a heavy object, initially diagnosed as torticollis, at an outside facility. On examination, she appeared ill and met the criteria for sepsis. Blood cultures confirmed MRSA. She developed hypotension, and an MRI of the brain and cervical spine revealed leptomeningeal enhancement and an epidural abscess. MRSA bacteremia, although common, can manifest in various forms. While it typically occurs in patients with identifiable risk factors, our patient had none. Identifying the source of bacteremia is crucial, as effective treatment requires both source control and antibiotic therapy. Given MRSA’s high morbidity and mortality, a thorough and rigorous approach to assessment and management is essential.

## Introduction

*Staphylococcus aureus* is a gram-positive, coagulase-positive organism that typically forms clusters. Methicillin-resistant *Staphylococcus aureus* (MRSA) is a strain of *S. aureus* resistant to oxacillin. MRSA infections can be classified as either hospital-acquired or community-acquired. While there has been a decline in hospital-acquired MRSA infections, community-acquired MRSA infections have been rising [1,2]. MRSA infections are associated with increased morbidity, mortality, and prolonged hospital stays [2]. Risk factors for MRSA include prolonged hospitalization, recent antibiotic use, MRSA colonization, invasive procedures, prostheses, HIV infection, intravenous drug use, and open skin wounds [1,2]. MRSA can cause disseminated infections, leading to bacteremia and seeding in various organs such as the heart, lungs, bones, and joints [1]. The infection typically spreads hematogenously but may also extend directly from adjacent tissues [1]. Effective management of MRSA infections requires source control, which can be challenging when the source of the bacteremia is unclear. We present a case of disseminated MRSA infection that necessitated a complex diagnostic approach, ultimately identifying the cervical epidural space as the probable source.

## Case presentation

A 63-year-old female with no significant past medical history presented with a two-day history of confusion and altered mental status. The patient’s family reported that she had become very drowsy and somnolent, with incoherent speech. They also noted a poor appetite. These symptoms began two days prior and progressed gradually. The family mentioned that the patient had developed neck stiffness after lifting a heavy load about four days before her current presentation. She had been seen at two different healthcare facilities for neck stiffness and was diagnosed with torticollis. She had been prescribed naproxen, hydrocodone, methylprednisolone, and cyclobenzaprine. Her husband reported that she started feeling “weird” after beginning these medications. The family denied any history of fevers, shortness of breath, cough, abdominal pain, diarrhea, nausea, vomiting, or dysuria. They had not noticed any skin rashes or joint swelling.

On presentation, the patient had tachycardia, tachypnea, and a fever of 101.6 °F. A physical examination revealed that she appeared ill and drowsy but was arousable to verbal stimuli. She was only oriented toward herself. Her neck was laterally rotated and stiff. Kernig’s and Brudzinski’s signs were negative. Her lungs were clear on auscultation, and her abdomen was soft, non-tender, and non-distended. The skin examination showed no track marks, petechiae, splinter hemorrhages, or Osler nodes.

Initial lab investigations revealed a WBC count of 8,700/ml with 21% bands, hemoglobin of 12.0 g/dL, and platelets of 100k/ml. Electrolytes were grossly unremarkable except for a creatinine level of 1.15 g/dL. The patient had a lactic acid level of 4.5 mmol/L. Urinalysis was positive for leukocyte esterase, with RBCs at 5/hpf and WBCs at 30/hpf. The urine drug screen was unremarkable (Table 1).

**Table 1:** Laboratory workup for the patient

Variable	Labs	Reference range
White blood cells (cells/mL)	8,700	4.5-11.0
Hemoglobin (g/dL)	12.0	12.0-16.0
Platelets (10^3^/mL)	100	150-400
Creatinine (g/dL)	1.15	0.5-1.1
Lactic acid (mmol/L)	4.5	<2

A chest X-ray revealed patchy bilateral infiltrates. A CT of the head was negative for any acute intracranial abnormalities. The patient met the criteria for severe sepsis. Likely sources of infection considered included pneumonia and UTI, and, given the neck stiffness, meningitis was also a concern. Broad-spectrum antimicrobials, including vancomycin, ceftriaxone, ampicillin, and acyclovir, were initiated. Unfortunately, a lumbar puncture could not be performed on admission due to logistical reasons.

Upon reevaluation, the patient had become hypotensive and hypoxic, requiring high levels of oxygen and exhibiting respiratory distress. She was admitted to the ICU and eventually intubated. Blood cultures, as well as cultures from urine and respiratory specimens, returned positive for MRSA, indicating a disseminated infection. The nucleic acid amplification test was positive for* S. aureus *with detectable mec A/C and MREJ genes. Efforts to identify the source of MRSA bacteremia were initiated. In the context of neck stiffness, an MRI of the cervical spine was performed, which showed leptomeningeal enhancement. An MRI of the brain revealed left transverse sinus, left sigmoid sinus, and left jugular thrombosis. CT of the chest, abdomen, and pelvis demonstrated bilateral pneumonia and bilateral pyelonephritis. An initial transthoracic echocardiogram performed on the first day of admission did not reveal any valvular vegetation. Based on the microbiological data, antibiotics were narrowed down to vancomycin (Table 2).

**Table 2:** Blood culture susceptibility report MRSA, methicillin-resistant* Staphylococcus aureus*

Antibiotic	Susceptibility to MRSA
Clindamycin	Susceptible
Erythromycin	Resistant
Oxacillin	Resistant
Penicillin	Intrinsic or inducible resistance
Rifampin	Susceptible
Tetracycline	Susceptible
Trimethoprim-sulfamethoxazole	Susceptible
Vancomycin	Susceptible

**Figure 1:**
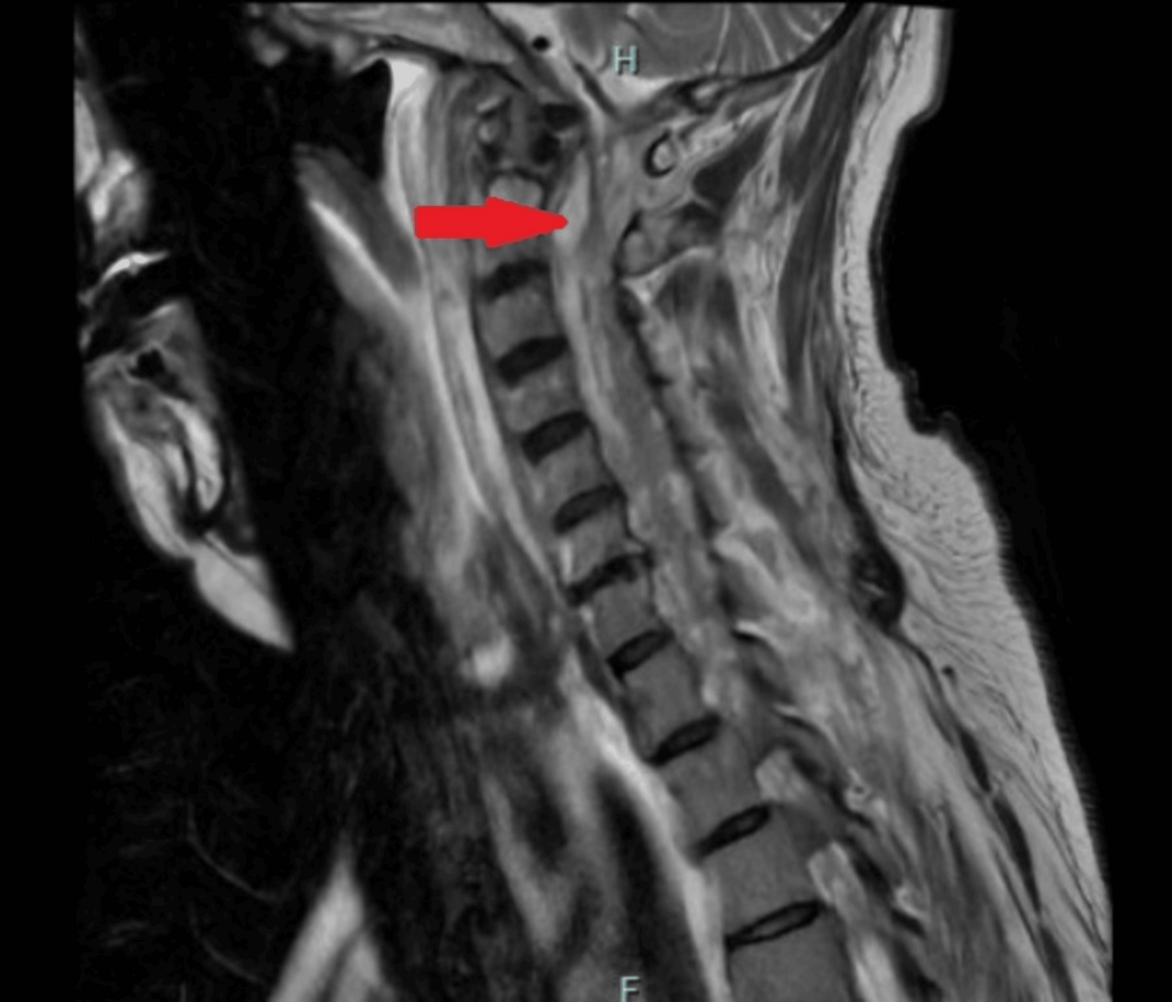
MRI of the cervical and thoracic spine (sagittal view) The MRI shows a hyperintense signal in the epidural space (arrow), consistent with an epidural abscess.

The patient’s subsequent blood cultures remained positive despite appropriate antibiotic treatment. Consequently, the infectious diseases team recommended adding intravenous ceftaroline. The source of the MRSA bacteremia remained unclear. Once the patient was hemodynamically stable, a transesophageal echocardiogram was performed on day 6 of admission, which was negative for vegetation. A repeat MRI of the cervical and thoracic spine revealed an extensive spinal epidural abscess extending from C1 to T3 (Figure 1). Neurosurgery was consulted, and the patient underwent a laminectomy from C1 to T3 and evacuation of the epidural abscess. Intraoperative body fluid cultures grew MRSA and *Staphylococcus* coagulase-negative species. Following source control, the patient’s clinical condition improved, and bacteremia was cleared one day post-operatively. The patient was discharged on intravenous daptomycin for six weeks via a peripherally inserted central catheter line.

## Discussion

MRSA bacteremia is a complex infection that can present in various ways. Initially associated with hospital-acquired infections, MRSA has seen a rise in community-acquired cases in recent years [3]. MRSA infections generally have higher mortality rates compared to methicillin-sensitive strains [4]. The outcomes of MRSA bacteremia are particularly severe due to decreased vancomycin efficacy in these cases [1]. Traditional risk factors for MRSA infection include prolonged hospitalization, especially in ICUs, recent antibiotic use, MRSA colonization, invasive procedures, prosthetic devices, HIV infection, intravenous drug use, and open skin wounds [1,2]. Our patient’s lack of identifiable risk factors makes this case particularly intriguing.

Identifying the source of MRSA bacteremia is crucial for effective source control. Although initial imaging and clinical symptoms suggested meningitis, MRSA meningitis is rare and usually not the primary cause of bacteremia [5]. Thus, we suspected that meningitis might be a manifestation of disseminated bacteremia rather than the primary infection source. This led to a repeat MRI, which revealed an extensive epidural abscess. Spinal epidural abscesses are uncommon but can lead to severe complications like spinal cord compression and paralysis [6]. Early spinal decompression and appropriate antibiotic therapy are critical in such cases [6].

This case underscores the importance of identifying common MRSA infection sites, such as skin/soft tissues (cellulitis and abscesses), bones and joints (osteomyelitis), lungs (pneumonia and abscesses), and heart valves (infective endocarditis) [1]. Diagnostic samples from suspected infection sources, including blood, sputum, urine, and wound scrapings, should be analyzed promptly, and treatment should include both source control and intravenous antibiotics [1].

For MRSA bacteremia, source control remains essential alongside culture-directed antibiotic therapy. The choice of antibiotic depends on the type of infection, local resistance patterns, drug availability, side effects, and patient-specific factors. Vancomycin and daptomycin are typically preferred for MRSA infections. In challenging cases, such as persistent bacteremia beyond 72 hours or unclear infection sources, a combination of antibiotics may be recommended, including daptomycin plus ceftaroline, vancomycin plus ceftaroline, daptomycin plus trimethoprim-sulfamethoxazole, or ceftaroline plus trimethoprim-sulfamethoxazole [7].

For our patient, surgical intervention to evacuate the abscess and a prolonged course of antibiotics led to significant improvement and a successful treatment response.

## Conclusions

Whenever patients present with MRSA bacteremia, a comprehensive physical examination and diagnostic workup are essential to identify the infection’s source. While spinal epidural abscesses are rare, they should be considered if more common infection sites have been ruled out. Clinicians must maintain a high index of suspicion in such cases to ensure accurate diagnosis and effective treatment.
